# Correlation of viral loads in disease transmission could affect early estimates of the reproduction number

**DOI:** 10.1098/rsif.2022.0827

**Published:** 2023-05-03

**Authors:** Thomas Harris, Nicholas Geard, Cameron Zachreson

**Affiliations:** School of Computing and Information Systems, The University of Melbourne, Parkville, Victoria, Australia

**Keywords:** reproduction number, multi-scale model, acute virus, epidemiology, viral load

## Abstract

Early estimates of the transmission properties of a newly emerged pathogen are critical to an effective public health response, and are often based on limited outbreak data. Here, we use simulations to investigate how correlations between the viral load of cases in transmission chains can affect estimates of these fundamental transmission properties. Our computational model simulates a disease transmission mechanism in which the viral load of the infector at the time of transmission influences the infectiousness of the infectee. These correlations in transmission pairs produce a population-level convergence process during which the distributions of initial viral loads in each subsequent generation converge to a steady state. We find that outbreaks arising from index cases with low initial viral loads give rise to early estimates of transmission properties that could be misleading. These findings demonstrate the potential for transmission mechanisms to affect estimates of the transmission properties of newly emerged viruses in ways that could be operationally significant to a public health response.

## 1. Introduction

Infectious disease outbreaks have dramatic impacts on communities. Accurate estimates of a pathogen’s transmission properties, such as the serial interval and basic reproduction number, are critical to inform the design of appropriate control measures. These properties are typically estimated based on outbreak data; therefore, estimation for a newly emerged pathogen can be challenging because limited data are available [1]. Estimates of these quantities are subject to multiple potential biases, with some biases stemming from the methods used to collect outbreak data and others arising from the statistical methods used for parameter estimation [1,2]. Failure to account for the ways these estimates can be misleading can lead to inaccurate conclusions around the scale of the threat posed by a particular pathogen, and result in subsequent public health responses that are poorly calibrated to the risk a community faces.

The basic reproduction number, *R*_0_, is defined as the average number of secondary cases produced by a single *typical* infected individual in an otherwise susceptible population. The effective reproduction number, *R*_*t*_, is the time-dependent number of secondary cases produced by a typical infectious case at time *t* after the introduction of the pathogen. Theoretically, the parameters *R*_0_ and *R*_*t*_ are approximately equivalent during the early exponential growth of a pathogen within a large population of susceptible hosts. Numerous methods have been developed for estimating *R*_*t*_ at some time *t* in an ongoing outbreak [2–6], and these are often used to estimate *R*_0_ based on early outbreak data. Cori *et al.* [4] describe a method for estimating *R*_*t*_ that uses infection incidence (number of new detected infections per day) up to some time *t* and knowledge of the serial interval (the time between the onset of symptoms in an infector and infectee transmission pair). This approach has been used in the analysis of various outbreaks [7–9] and has been extended to account for heterogeneity in transmission, capturing differences in transmission potential between a discrete set of groups in a population [6].

Several studies have investigated potential sources of bias that can affect estimates of the reproduction number [1–3]. For example, incorrectly including imported cases in local case counts exaggerates the number of secondary cases attributed to local spread and can lead to overestimates of the transmissibility of a pathogen [1,2]. Similarly, these studies identified over-representation of cases with a lower or higher intrinsic transmission rate early in an outbreak [1] and appropriate selection of serial interval data [2] as important potential sources of bias to consider in establishing accurate estimates of the reproduction number. While these observational sources of bias can be accounted for through enhanced surveillance, another complicating factor is the natural heterogeneity in the disease progression of each individual. For example, diverse expressions of illness in infected individuals can affect the capacity to understand transmission of a disease [6].

For viruses, the viral load dynamics within hosts have been found to vary between infected individuals. The reason for this heterogeneity in viral load dynamics is not well understood. One possible mechanism that could contribute is variation in the initial viral load, the viral quantity transmitted from a donor host that initiates an infection in an exposed recipient [10–15]. Key aspects of viral load dynamics, such as duration of infection and peak viral load, are impacted by the initial viral load [16,17]. Furthermore, a positive relationship has been observed between a host’s viral load and their infectiousness [18,19]. From a modelling perspective, there has been discussion on the strong intuitive connection between pathogen load and infectiousness, while also acknowledging the possible complications introduced by varying symptom development and host behaviour [18].

In this work, we form and investigate a hypothesis based on the well-established findings discussed above, namely that:

1. higher viral load corresponds to increased probability of transmission to a new susceptible host (given contact), and
2. being exposed to higher levels of virus produces higher peak viral loads because of the larger initiating quantity.

Intuitively, these two results indicate the potential for correlations to exist in transmission chains, i.e. that a transmission chain initiated with a highly infectious index case may generate higher-than-average transmission rates, and vice versa. Our hypothesis is that such correlations of within-host viral dynamics between transmission pairs could affect estimates of transmission properties produced during the early stages of an outbreak.

To investigate this hypothesis, we designed and implemented a multi-scale model of infectious disease transmission to capture correlations of within-host viral load dynamics and their effects on estimation of transmission properties. Multi-scale models have increasingly been used to capture infectious disease spread [20–22]. These models typically capture disease dynamics at both a within-host scale (i.e. how an infection progresses inside a single individual) and a between-host scale (i.e. how the infection is transmitted between multiple individuals of a host population) [20]. These types of models have been used to help address open questions relating to a variety of infectious diseases, such as influenza A [23] and Ebola [24]. Importantly, multi-scale modelling allows for explicit representation of individual-level processes, such as host viral load, and the linking mechanism that connects these processes to population-level spread dynamics.

Our model describes a general acute respiratory virus spreading in a population. We assume three key relationships in the transmission of the virus:

1. Host infectiousness increases with viral load.
2. The trajectory of the host viral load over the course of infection is related to the initiating quantity of virus.
3. Recipient initial viral load increases with the donor viral load at the time of transmission

Assumption 1 is supported by experimental studies (e.g. [19]) and has been used in previous simulation studies [18,22,25]. Similarly, assumption 2 is supported by experimental studies and several existing within-host models of pathogens [10,16,17]. While we are not aware of any direct evidence supporting assumption 3, this assumption is consistent with studies of SARS (e.g. [26]) and tuberculosis (e.g. [27]) that suggest the inoculum dose size (the quantity of pathogen presented to a susceptible host), has a positive relationship with the pathogen load measured after infection. Assumption 3 has also been used by previous simulation studies [24,28].

We investigate how these mechanisms of viral transmission produce dynamics that violate core assumptions typically made when estimating reproduction numbers. Namely, that the reproduction number does not vary during the early stages of an outbreak. We found that correlation of viral loads in transmission pairs produces a population-level convergence process, during which the distribution of initial viral loads in each subsequent generation converges to a steady state. Thus, outbreaks arising from index cases with low initial viral loads give rise to early estimates of transmission properties that differ substantially from their converged values. Our work demonstrates how biologically feasible mechanisms of viral transmission can complicate the interpretation of epidemiological parameters estimated through population-level case data.

## 2. Methods

In this section, we describe:

— the multi-scale model used to simulate disease spread in a host population,
— the analytical method we developed to quantify the rate at which correlations in viral load change over the course of an outbreak, and
— the methods we used to estimate reproduction numbers and serial intervals based on simulated outbreak data.

At the end of the section, we outline the three main experiments conducted in this study.

### 2.1. Multi-scale model of disease dynamics

#### 2.1.1. Model of case progression and virus transmission between hosts

Between-host disease transmission is described by an agent-based model (ABM). This modelling approach is well suited to capturing infectious disease spread, because it allows for the explicit simulation of complex interaction behaviour and heterogeneity between individuals. ABMs are used across the infectious disease multi-scale modelling literature [20].

**Figure 1.**
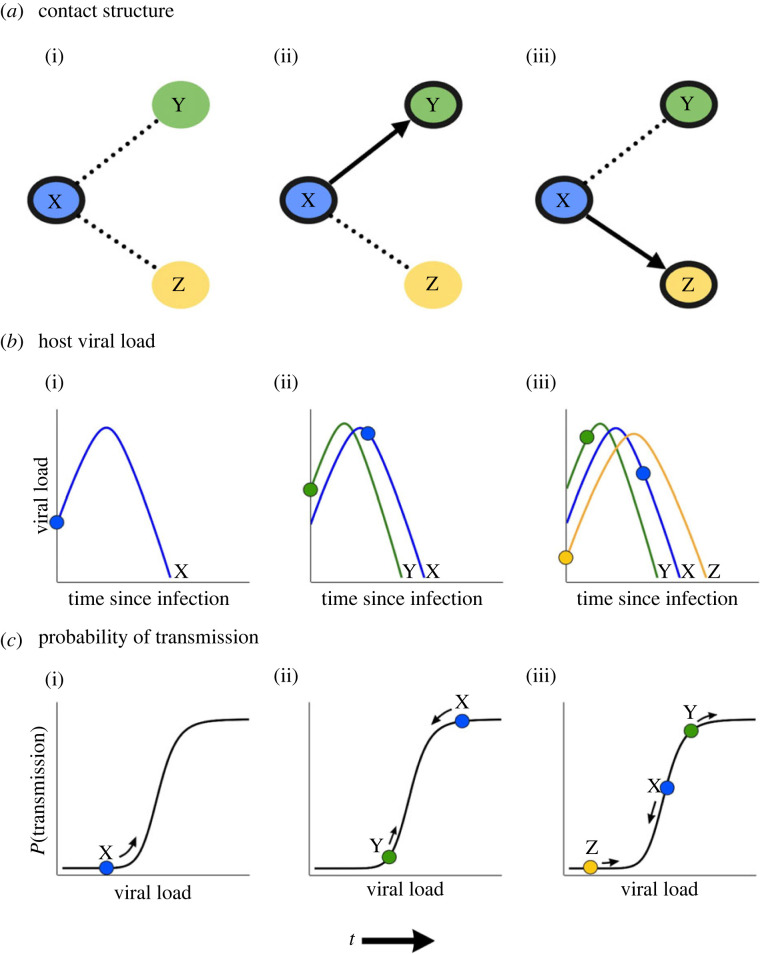
Schematic of the mechanistic model of disease transmission. The viral load trajectory of each secondary case depends on the viral load of the primary case at the time of transmission. Here, the primary case X (i) infects two secondary cases, Y (ii) and Z (iii). The viral load trajectories of cases Y and Z depend on the viral load of the primary case X at the time of transmission (*b*), which also influences the probability of transmission occurring via the sigmoidal mapping in (*c*).

Our ABM simulates random interactions between individuals in a large, well-mixed population. At any point in time, an agent exists in one of four states, which correspond to the compartments of a susceptible–exposed–infectious–recovered (SEIR) compartmental model (see electronic supplementary material, S1). We simulate stochastic disease transmission in an infinitely large population. At each discrete time step, the probability of transmission from each infectious individual is evaluated. If transmission is successful, a new infected agent is produced with properties determined by the viral load of the donor (figure 1). The within-host model of viral load as a function of time determines the time spent in the ‘exposed’ state, the infectious period and the time until recovery. There are 12 time steps (*S*_*d*_ = 12) in each simulation day (i.e. interactions between individuals are simulated 12 times across a day). We chose this contact process for two reasons: (i) to represent disease transmission in an ‘infinite’ population, such that any errors in estimation of transmission properties could be attributed to the transmission mechanism, rather than the contact process, and (ii) to contain the simulation time. We tested the convergence of the secondary case and serial interval distributions produced by the model for decreasing time-step width, and chose *S*_*d*_ = 12 days to obtain an acceptable balance between model runtime and precision (see electronic supplementary material, S13).

To compute the viral load as a function of time since infection, we define a within-host model of disease progression (see §2.1.2) which guides transitions between compartments. As mentioned above, infected individuals are initially ‘exposed’, that is, they cannot transmit infection. As their viral load increases past a defined threshold *V*_*T*_ (here set to *V*_*T*_ = 13431.67—see electronic supplementary material, S1), they transition from the ‘exposed’ to ‘infectious’ state. After passing through a peak, the infected individual’s viral load gradually decreases. When the viral load decreases past *V*_*T*_, the individual is no longer infectious, and has transitioned to the ‘recovered’ state.

#### 2.1.2. Model of within-host viral dynamics

To simulate viral load dynamics within individual hosts, we chose a mathematical model developed by Steinmeyer *et al.* [28] for the dynamics of acute respiratory infections similar to those caused by influenza or SARS. These types of models have been applied extensively to within-host viral dynamics, and typically include features associated with self-limiting viral replication as well as the activity of the host immune response [10,28–30].

The system of ordinary differential equations (ODEs) that describe the within-host viral dynamics is given by

2.1 $$\frac{dV}{dt}=rV-V\left(\frac{rV}{K_{v}}+k_{I}I+k_{N}N+k_{P}P\right),$$

2.2 $$\frac{dI}{dt}=a_{I}V+b_{I}\left(1-\frac{I}{K_{I}}\right),$$

2.3 $$\frac{dN}{dt}=a_{N}V\Theta (t-\tau_{N})-d_{N}N$$

2.4 $$\mathrm{and}\;\;\frac{dP}{dt}=a_{P}VP+cN(t-\tau_{P}),$$

where *V* is the number of viral particles, *I* is the strength of the innate immunity, *N* is the number of non-specific memory cells, *P* is the number of specific memory cells, *t* is the time since initial infection, *r* is the viral replication rate, *K* is the carrying capacity (population of each cell type, denoted by subscript letter, that can be supported in the body), *a* is the growth rate per virion relevant to each immune defence (denoted by subscript letter), *k* is the rate of viral removal relevant to each immune defence (denoted by subscript letter), *τ* represents the time delay after which the development of non-specific memory cells (*τ*_*N*_) or specific memory cells (*τ*_*P*_) can occur, *b*_*I*_ is the constant rate of growth for the innate immunity, *c* is the specific memory cell growth rate proportional to non-specific memory cells and *d*_*N*_ is the non-specific memory cell decay rate. Θ describes a Heaviside step function given by

2.5 $$\Theta (t)=\left\{ \begin{array}{cc} 0,\; & \mathrm{if}\;t<0\;\\ 1,\; & \mathrm{if}\;t\geq 0\; \end{array}.\right.$$

The specific parameter values chosen in our model as implemented are provided in electronic supplementary material, S1. To introduce individual-level heterogeneity of within-host immune response, carrying capacity parameters (*K*_*v*_ and *K*_*I*_) and the rates of viral removal relevant to each immune defence (*k*_*I*_, *k*_*N*_ and *k*_*P*_) were sampled from gamma distributions for each infected individual (see electronic supplementary material, S1). Taken together, this choice of model and accompanying parameters describe within-host viral load dynamics that depend substantially on the initial viral load (see electronic supplementary material, S2). Specifically, higher initial viral loads correspond with a faster viral proliferation and a higher peak load, but also produce a stronger immune response and a faster recovery. See [28] for a more detailed description of the within-host model used here.

#### 2.1.3. Modelling correlations in viral load between cases in transmission chains

For each interaction between an infectious donor and susceptible recipient host, the donor viral load at transmission determines two key values: infectiousness of the donor and the initial viral load of the recipient. At the time of exposure, the donor viral load (*V*_*d*_) translates to a probability of transmission, given exposure (*P*_trans_) via a sigmoidal mapping (figure 1). This mapping is given by

2.6 $$P_{\mathrm{trans}}=\frac{c_{1}V_{d}^{\zeta }}{V_{d}^{\zeta }+c_{2}^{\zeta }},$$

where *c*_1_ is the maximum *P*_trans_, *c*_2_ is the donor viral load at which *P*_trans_ is half-maximal and *ζ* is a slope parameter characterizing the steepness of the transition from low to high infectiousness. The specific parameter values chosen in our model as implemented are provided in electronic supplementary material, S3. The sigmoidal shape was chosen based on experimental results suggesting this kind of relationship may describe transmission likelihood [18,27] and its use in other models of disease transmission [22,31].

Second, given transmission has occurred, the donor viral load (*V*_*d*_) translates to a value for the initial viral load that initiates infection in the recipient (*V*_*R*_(0)) also via a sigmoidal mapping,

2.7 $$V_{R}(0)=\frac{d_{1}V_{d}^{\kappa }}{V_{d}^{\kappa }+d_{2}^{\kappa }},$$

where *d*_1_ is the maximum *V*_*R*_(0), *d*_2_ is the donor viral load at which *V*_*R*_(0) is half-maximal, and *κ* is a slope parameter characterizing the steepness of the transition from low to high *V*_*R*_(0). The specific parameter values chosen in our model as implemented are provided in electronic supplementary material, S4. Though it is not supported by any direct empirical evidence we are aware of, we chose the sigmoid function in equation (2.7) because there is evidence suggesting this functional form could probably describe the relationship between pathogen load and the likelihood of transmission, a closely related property [18,27].

### 2.2. Quantifying the convergence of transmission dynamics

In this model of disease transmission, the initial viral load of a host determines the trajectory of their infectiousness over time. Therefore, at any time *t* during a simulated outbreak, the distribution of initial viral loads P(V(0) | t) of the cases currently infected provides a useful summary statistic for the underlying dynamics. By examining how *P*(*V*(0)) changes as a function of time, we observe how the transmission dynamics converge to a stable condition that does not depend on the initial state.

To quantify this convergence process, we define a tolerance of *δ* = 0.0475 and perform a Kolmogorov–Smirnov (KS) test to compute the KS statistic comparing P(V(0) | g) for each subsequent generation *g* of infectious cases (note that here we compute the distribution of initial viral loads for each generation, rather than for a snapshot in time). After the generation *g*_*c*_ when the KS statistic KS(*P*(*V*(0)|*g*_*c*_), *P*(*V*(0)|*g*_*c*_ + 1)) < *δ* and KS(*P*(*V*(0)|*g*_*c*_), *P*(*V*(0)|*g*_*c*_ + 2)) < *δ*, we consider the system’s dynamics to be stable. Stability means that the properties of the transmission dynamics will no longer change substantially as time goes on, as long as the infected and recovered populations are much smaller than the susceptible population.

### 2.3. Estimation of transmission properties

To understand specifically how the transmission process of this model could affect early outbreak data, we investigated how the initial viral load of the index case (*V*(0)_index_) affected estimation of the serial interval and the reproduction number across the early portion of an outbreak. The methods outlined in this section are further described in electronic supplementary material, S5.

#### 2.3.1. Estimating the serial interval

Because our model does not explicitly represent symptom expression, we used the time between infection and peak viral load as a surrogate for the incubation period (the time between infection and symptom onset). We assumed that symptom onset was always observed and reported on the day at which symptom onset occurred. We also assumed all transmission pairs could be established immediately without error, once the symptom onset of the infectee had occurred. Serial intervals were then determined by measuring the difference between symptom onset times of donor and recipient pairs.

As each outbreak continued to grow after each observation time, we accounted for the effects of right truncation in the data [32]. We corrected for these effects by limiting the data to transmission pairs where (i) the donor is no longer infectious, and (ii) all secondary cases produced by the donor have reached symptom onset.

We fitted a gamma distribution to the set of serial intervals observed. We then performed parametric bootstrapping to estimate the uncertainty associated with the fitted distribution. This process generated a set of *N*_*B*_ = 100 bootstrapped instances (sets of shape (*α*) and rate (*β*) parameters). From this set, we were able to establish the mean and 95% confidence intervals (CIs) of the fitted gamma parameters. We used this bootstrapping approach to characterize the uncertainty in the serial interval and propagate this uncertainty to the reproduction number estimation process, as has been done effectively using Markov chain Monte Carlo (MCMC) methods elsewhere [2].

#### 2.3.2. Estimating *R*_0_

The methods for estimating the reproduction number often target *R*_*t*_—the average number of secondary cases produced by an infected individual in a population that may have some level of immunity, at some time *t* in an ongoing outbreak [2–6]. Using our model, we simulated an early outbreak time period where depletion of susceptible individuals is yet to occur, and individuals have no existing immunity. With these assumptions, we estimated the basic reproduction number (*R*_0_) by estimating *R*_*t*_ during the early exponential phase of outbreak growth.

After estimating the serial interval distribution for a particular outbreak, we used a method to estimate *R*_0_ similar to the Bayesian technique developed by Cori *et al.* [4].

The process for estimation of *R*_0_ at some time *t* in a given outbreak can be described by four key steps:

— For each bootstrapped instance of the serial interval distribution describing this outbreak, we discretized the continuous gamma distribution. This discretization produced a discrete probability mass function *ω*_*s*_, which described the likelihood of the serial interval being some integer number of days *s*.
— We computed the expectation of infectivity ($\Lambda_{j}$) for each day *j* in the period of days *t* − *τ* to *t*, where we assumed the reproduction number remains constant over this time period. The expectation of infectivity ($\Lambda_{j}$) on some day *j* is computed as
  2.8 $$\Lambda_{j}=\sum_{s=1}^{\;j}I_{\;j-s}\omega_{s},$$
  where *I*_*j*−*s*_ refers to case incidence on day *j* − *s*, and *ω*_*s*_ refers to the discrete probability mass function.
— We used the Bayesian framework detailed in [4] to describe the posterior distribution of the reproduction number at time *t* as a gamma distribution with shape (*α*) and scale (*β*) parameters,
  2.9 $$\alpha =a+\sum_{k=t-\tau }^{t}I_{k}$$
  and
  2.10 $$\beta =\frac{1}{\frac{1}{b}+\sum_{k=t-\tau }^{t}\Lambda_{k}},$$
  where *a* and *b* refer to the shape and scale parameters, respectively, of the assumed gamma prior distribution. Here, we chose *a* and *b* so that the mean and standard deviation of the prior distribution are both equal to five (*a* = 1 and *b* = 5), similar to other studies [2,4]. These parameter values ensured the prior distribution was relatively uninformative and conservative.
— Finally, we sampled the posterior distribution of *R*_0_ *N*_*S*_ = 100 times to produce a set of estimates of the reproduction number at time *t*. We pooled over all the sets of *R*_0_ estimates produced from the bootstrapped instances of the serial interval distribution, establishing a set of *N*_*S*_ × *N*_*B*_ estimates of *R*_0_ at time *t* in a given outbreak.

See [4] for a more detailed description of the Bayesian framework used here.

Because we used a stochastic model of disease transmission, we simulated multiple outbreaks for each scenario to characterize the typical behaviour of the system for each set of conditions. To analyse a set *O* of outbreaks for a scenario, we computed the mean *R*_0_ estimate across *O* for each day *d* after the first case is detected. Specifically, we pooled the *N*_*S*_ × *N*_*B*_ *R*_0_ estimates produced from each outbreak simulation, observed for *d* days after the first case, to create a set of *R*_0_ estimates of size |*O*| × *N*_*S*_ × *N*_*B*_. We then computed the mean of this set of *R*_0_ estimates as a global summary statistic, which we denote as 〈*R*_0_〉_*d*_.

#### 2.3.3. Measuring *R*_0_ estimation bias and confidence

We measured the magnitude of the bias and the certainty associated with the distribution of *R*_0_ estimates produced at each day *d* in a single outbreak simulation, *i*, in a set of outbreak simulations, *O*.

To quantify the bias, we computed how the maximum likelihood estimate (MLE) of the distribution computed using observations up to day *d* compares to the ‘true’ *R*_0_, which we computed from the secondary case distributions at day 50 across the whole outbreak set *O*,

2.11 $$\mathrm{Bias}(i,d)=\frac{\left|{\left\langle R_{0}\right\rangle }_{i,d}-R_{0}^{\mathrm{true}}\right|}{R_{0}^{\mathrm{true}}},$$

where 〈*R*_0_〉_*i*,*d*_ is the maximum likelihood *R*_0_ estimate on day *d* for simulation *i* and $R_{0}^{\mathrm{true}}$ is the ‘true’ *R*_0_. To calculate $R_{0}^{\mathrm{true}}$, we measured the mean of the secondary case distribution at day 50 for each outbreak in the set *O*. The average of this set of means was then defined as $R_{0}^{\mathrm{true}}$.

We also measured the relative confidence in each distribution by computing the ratio of the maximum likelihood estimate to the width of the 95% CI,

2.12 $$\mathrm{Confidence}(i,d)=\frac{{\left\langle R_{0}\right\rangle }_{i,d}}{q_{0.975}-q_{0.025}}\;,$$

where 〈*R*_0_〉_*i*,*d*_ is the maximum likelihood *R*_0_ estimate on day *d* for simulation *i*, and *q*_0.975_ − *q*_0.025_ is width of the 95% CI.

To summarize the bias and confidence for some day *d* across a simulated set of outbreaks, *O*, we computed

2.13 $$\mathrm{Bia}s^{\prime }(d)=\frac{\sum_{i=1}^{\left|O\right|}\mathrm{Bias}(i,d)}{\left|O\right|}\;\;$$

and

2.14 $$\mathrm{Confidenc}e^{\prime }(d)=\frac{\sum_{i=1}^{\left|O\right|}\mathrm{Confidence}(i,d)}{\left|O\right|}.\;$$

### 2.4. Experimental design

#### 2.4.1. Effect of the initial viral load of the index case on outbreak growth

To understand how the initial viral load of the index case affected early outbreak development, we varied *V*(0)_index_ and measured the effect on case incidence during the early stages of outbreaks. We investigated three initial viral load values—*V*(0)_index_ = 4.5, 45 and 450—and initialized 200 outbreaks with each setting. This number of simulations for each *V*(0)_index_ setting ensured we accurately captured the range of behaviour that could be produced under each setting. For each simulated outbreak, we measured the case incidence over the first 50 days after the first detected case. We disregarded and reran simulations in which the outbreak dies out (i.e. case prevalence reaches 0) before 50 days. Cases were detected perfectly at symptom onset (approximated by the time of peak viral load, *t*_Vmax_). We scaled the sigmoid function describing transmission potential as a function of viral load, *P*_trans_ (see equation (2.6)), such that the $R_{0}^{\mathrm{true}}\approx 2$ (see electronic supplementary material, S3). This scaling of the transmission rate meant that simulating uninhibited growth across all index case viral loads was computationally feasible. Note that this differs from the scaling magnitude used in the third experiment where a larger $R_{0}^{\mathrm{true}}$ was chosen to more clearly demonstrate the potential for misleading parameter estimates.

#### 2.4.2. Effect of the initial viral load of the index case on convergence of transmission dynamics

To understand the relationship between *V*(0)_index_ and the convergence of transmission dynamics in an outbreak, we varied *V*(0)_index_ and measured the effect on the rate of convergence in the distribution of initial viral loads (see §2.2). We investigated five initial viral load values—$V{\left(0\right)}_{\mathrm{index}}=4.5,14.2,45,142\;\mathrm{and}\;450$—and initialized 200 outbreaks under each setting. We simulated each outbreak until 10 000 cases were recorded. We disregarded and reran outbreaks which did not meet this final outbreak size in order to ensure a well-sampled distribution of initial viral loads. We measured the rate of convergence using the described method (see §2.2). We pooled the initial viral load data for each generation across simulations to capture the typical behaviour of the system. As in the first experiment, we scaled *P*_trans_ (see equation (2.6)) such that $R_{0}^{\mathrm{true}}\approx 2$ (see electronic supplementary material, S3).

#### 2.4.3. Effect of the initial viral load of the index case on estimation of transmission properties

In this experiment, we aimed to establish how any link between *V*(0)_index_ and the early outbreak dynamics determined in the convergence of transmission dynamics analysis (§2.2), affected the estimation of transmission properties. We used two main parameter sets in this experiment. First, we investigated three *V*(0)_index_ values—$V{\left(0\right)}_{\mathrm{index}}=4.5,45\;\mathrm{and}\;450$—and scaled *P*_trans_ (see equation (2.6)) such that the $R_{0}^{\mathrm{true}}\approx 2$ (see electronic supplementary material, S3). Second, we investigated a low *V*(0)_index_ value—*V*(0)_index_ = 4.5—and scaled *P*_trans_ (see equation (2.6)) such that the $R_{0}^{\mathrm{true}}\approx 4$ (see electronic supplementary material, S3).

We initialized 200 outbreaks for each *V*(0)_index_ value and simulated each up to 50 days. We disregarded and reran simulations in which the outbreak dies out (i.e. case prevalence reaches 0) before 50 days. We applied the transmission property estimation methods described (§2.3) each day between a range of 26–50 days of each outbreak. We did not analyse the first 25 days, as the extremely limited case data meant the methods were unlikely to be reliable [4]. On each day, the data consists only of the serial intervals (corrected for right truncation) and incidence of case symptom onsets observed up to that day. Each observation was designed to simulate how estimation would proceed when no previous sources of data are available (i.e. observing a novel pathogen).

We assumed model conditions that allowed us to assess *R*_0_. In particular, we assumed a large, well-mixed population that ensured susceptible depletion was not substantial early in an outbreak (electronic supplementary material, S15), and we assumed host behaviour was unchanged over the observed time frame. These assumptions meant the transmission dynamics were governed by the same set of parameters over the whole simulation. As such, we set the lower bound of the time window for computing the reproduction number (*t* − *τ*) to zero. This setting meant we analysed the entire outbreak observed up to time *t* when estimating *R*_0_ and the time window width (*τ*) would grow as *t* increased.

## 3. Results

### 3.1. Effect of the initial viral load of the index case on outbreak growth

**Figure 2.**
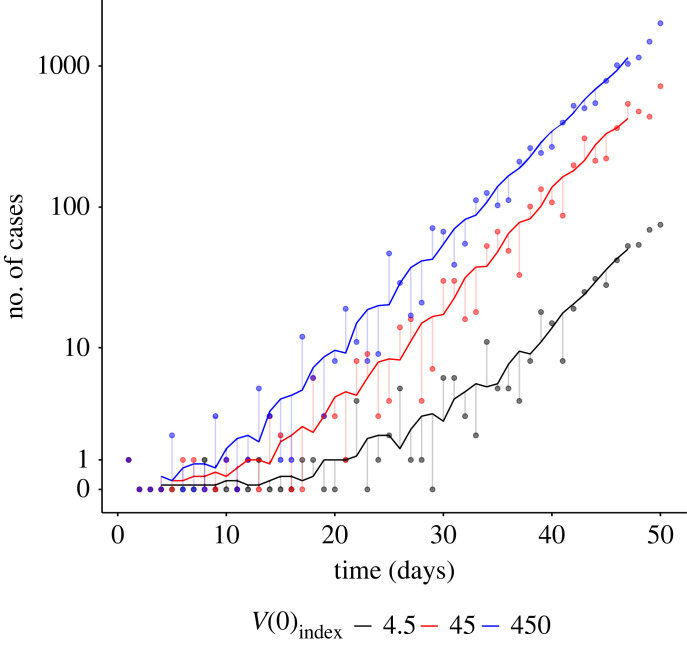
Time series plots of early disease incidence (number of newly detected cases) of outbreaks with different index case viral loads. The early rate at which an outbreak grows increases as the initial viral load of the index case (*V*(0)_index_) increases. The effect of the initial viral load of the index case on outbreak growth reflects a multi-generational correlation of infection dynamics between cases in a transmission chain, introduced by the transmission mechanism simulated by our model. Representative incidence trajectories shown here were chosen from a set (*N* = 200) based on a defined set of outbreak summary statistics (see electronic supplementary material, S6). The 7-day smoothed mean (solid line) is shown alongside the raw data (points).

Increasing the initial viral load of the index case (*V*(0)_index_) has a substantial effect on the case incidence trajectory in the first 50 days (figure 2). Under low *V*(0)_index_ settings, fewer cases emerge across the observed period and outbreaks typically grow at a slower rate. This result reflects the different infection dynamics of the index case between settings, and their effect on subsequent cases produced in the early generations of an outbreak.

We also observe a distinct periodicity to the growth of case incidence which produces a ‘sawtooth’ pattern in the data around the 7-day smoothed mean (figure 2). This pattern emerges from correlation in the timing of the peak viral load (*t*_*V*_max), the most infectious period for a case, between infections in transmission chains. This correlation introduces a periodic rise in new infections.

### 3.2. Effect of the initial viral load of the index case on convergence of transmission dynamics

**Figure 3.**
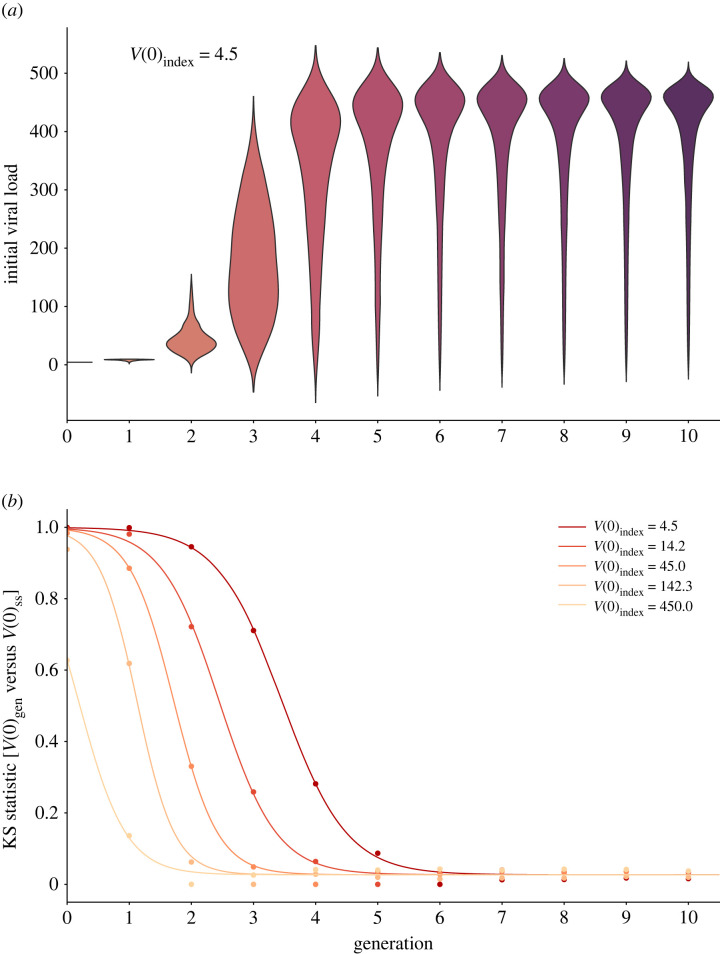
Dynamics of the distribution of initial viral loads as a function of contagion generation. The violin plots in subfigure (*a*) demonstrate how the distribution of initial viral loads over all infected cases evolves as the contagion spreads when the initial viral load of the index case is low (*V*(0)_index_ = 4.5). The trajectories of KS statistics in subfigure (*b*) show how the viral load distribution at each generation [*V*(0)_gen_] approaches a converged state [*V*(0)_SS_], occurring at some converged generation *g*_*c*_, with the rate of approach increasing with the initial viral load of the index case. Dots represent specific KS statistic values for each generation; solid lines represent fitted logistic curves, used for visualization purposes.

The rate at which the system converges is positively related to *V*(0)_index_ (figure 3*b*). That is, the number of generations required before system dynamics converge (*g*_*c*_) decreases as *V*(0)_index_ increases. This trend is most clear in the lowest *V*(0)_index_ simulated (*V*(0)_index_ = 4.5), where five to six generations of outbreak growth are required before the initial viral load distribution converges (figure 3*a*). The number of generations required for convergence, *g*_*c*_, is substantially smaller when the highest *V*(0)_index_ is simulated (*V*(0)_index_ = 450), where convergence occurs in two to three generations (see electronic supplementary material, S7). This occurs because our transmission model produces correlation between the viral load of an infectious individual at the time of transmission and the initial viral load of the secondary case.

Importantly, the relationship between *V*(0)_index_ and the convergence of transmission dynamics demonstrates that incidence data produced in the early generations of outbreaks may not be representative of future case dynamics. Furthermore, how representative these early cases are of the later dynamics of the system is related to the infection dynamics of the index case. In the following section, we investigate how this result relates to estimations of the serial interval and *R*_0_.

### 3.3. Effect of the initial viral load of the index case on estimation of transmission properties

#### 3.3.1. Estimating the serial interval

The mean estimate of the serial interval converges to a value of 4.2–4.3 days across the 50 days of observation, regardless of *V*(0)_index_ (see electronic supplementary material, S8). There is typically an overestimation of the serial interval early in the outbreak observation window in the scenarios with low *V*(0)_index_ (e.g. *V*(0)_index_ = 4.5). This overestimation is reflective of the longer incubation periods in infections with low initial viral loads (see electronic supplementary material, S2), which dominate these outbreaks in the early phase.

#### 3.3.2. Estimating *R*_0_

The mean estimate of the reproduction number, 〈*R*_0_〉_50_, converges to a value of 2–2.1 across the 50 days of observation, regardless of *V*(0)_index_ (see electronic supplementary material, S9). In the early period of observation (days 26–30), the central estimate (〈*R*_0_〉_*d*_, where *d* is between 26 and 30) of the scenarios with lower *V*(0)_index_ is typically an underestimate of the eventual converged value; for example, the lowest *V*(0)_index_ setting (*V*(0)_index_ = 4.5) produces 〈*R*_0_〉_*d*_ across days (*d*) 26–30 of between 1.7 and 1.8. The underestimation of *R*_0_ in these scenarios can be attributed to the fact that cases with lower initial viral loads will typically transmit to fewer individuals due to their lower peak viral load. As these cases typically dominate the early period of the scenarios with low *V*(0)_index_, lower transmission is observed.

We also estimated *R*_0_ for a low *V*(0)_index_ setting (*V*(0)_index_ = 4.5) when $R_{0}^{\mathrm{true}}$ (ground truth reproduction number in the converged state) is higher—$R_{0}^{\mathrm{true}}\approx$ 4. Simulating a larger $R_{0}^{\mathrm{true}}$ alongside our previous analysis allowed us to understand how the early instability of the transmission dynamics persists in a more infectious outbreak scenario. We increased $R_{0}^{\mathrm{true}}$ by applying a scaling factor of 2 to our definition of *P*_trans_ (see electronic supplementary material, S3).

**Figure 4.**
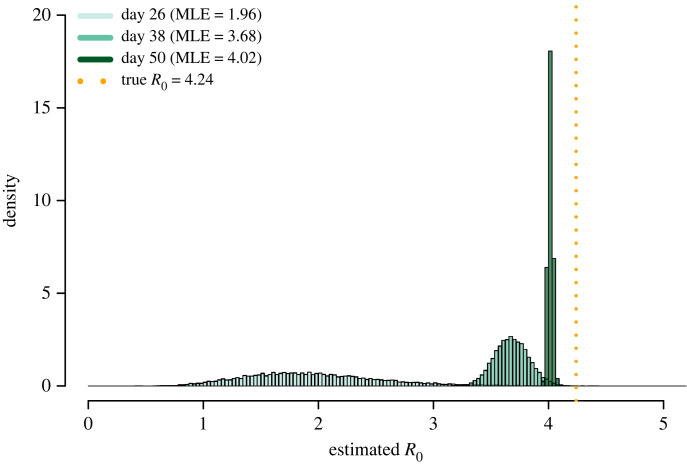
The posterior distribution of *R*_0_ at three time points (days 26, 38 and 50) for a single representative outbreak with a low *V*(0)_index_ (*V*(0)_index_ = 4.5). The central estimate of the posterior distribution increases over the course of the 50 day observation period of the outbreak, moving from 1.96 at day 26 up to 4.02 on day 50. As the contagion progresses, the estimate approaches the approximation of the true *R*_0_ ($R_{0}^{\mathrm{true}}$) of the system 4.24 (orange dashed line). The variance of the posterior decreases over the course of the outbreak, reflecting the increasing amount of case data becoming available and the increasing homogeneity of the infection dynamics among cases. The outbreak producing the data for this figure was selected as representative from a set (*N* = 200) based on a defined set of outbreak summary statistics (see electronic supplementary material, S6).

Comparing the effect of a higher transmission rate on the development of the outbreak, the magnitude of the difference between early (days 26–30) and later (days 45–50) estimates increases, but the number of days required for convergence remains similar (see electronic supplementary material, S10). Examining a representative outbreak with $R_{0}^{\mathrm{true}}\approx$ 4, we observe a substantial underestimation of *R*_0_ over the first 40 days of the outbreak (figure 4). The uncertainty in these estimates is initially high (day 26—95% CI: 0.968–3.356) and decreases across the observation period (day 38—95% CI: 3.389–3.983 and day 50—95% CI: 3.977–4.057). We note that at day 50 the estimated *R*_0_ (MLE = 4.02) is still marginally below the $R_{0}^{\mathrm{true}}=4.24$. This is due to assumptions relating to the observation of cases in our model (see electronic supplementary material, S15).

**Figure 5.**
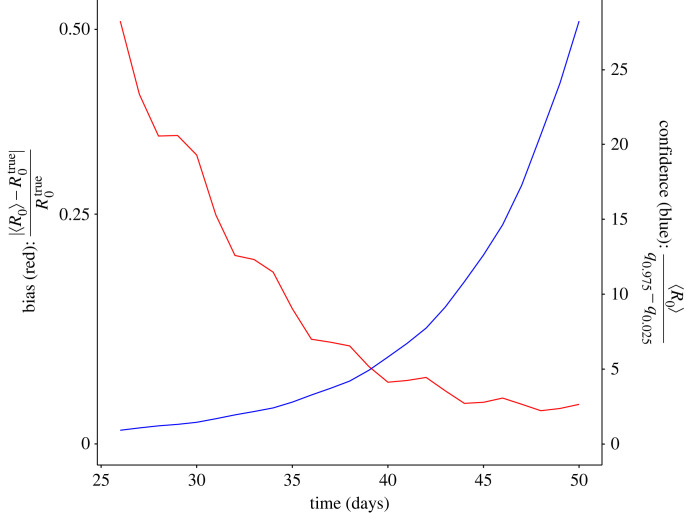
Relationship between relative bias and confidence when estimating the reproduction number over time. The bias (measured as the relative difference between the estimated and the true value of *R*_0_) decreases toward zero, as the initial conditions approach the relaxed state. As bias decreases, confidence increases (measured as the ratio of the estimated value and the 95% interquantile range), as the number of observed cases and similarity of the new cases increases.

We computed the mean bias and confidence (see equations (2.13) and 2.14) from the set of outbreak simulations at each day across our observation window (figure 5). The mean bias, Bias′, decreases over the course of the observation period, falling from Bias′ between 0.3 and 0.5 across days 26–30 to Bias′ ≈ 0 across days 40–50. This decrease reflects the convergence of the case dynamics that occurs across this period. The mean confidence, Confidence′, increases over the course of the observation period from Confidence′ ≈ 0 across days 26–30 to Confidence′ between 5 and 25 across days 40–50. This rise in Confidence′ is reflective of the increasing case count and the increasing homogeneity in the infection dynamics of newly infected individuals. Figure 4 demonstrates the magnitude of the bias and confidence when estimating the reproduction number for a single outbreak as a function of time.

## 4. Discussion

Understanding the transmissibility of a pathogen is critical for designing effective policies to reduce its spread in a community. Accurate estimation of transmission properties, such as *R*_0_, is crucial for establishing the scale of risk posed in future outbreaks. Producing accurate estimates of transmission properties with limited observed data is difficult, particularly when there is substantial heterogeneity in the disease characteristics of the infected population.

In this study, we demonstrated that assumptions made about viral transmission can lead to time-varying estimates of the serial interval and *R*_0_. We used a convergence analysis method, together with previously published serial interval and reproduction number estimation procedures, to demonstrate that early outbreak dynamics are substantially dependent on the initial viral load of the index case, *V*(0)_index_ (figures 2 and 3), and hence estimates of transmission properties based on observations in this period could be misleading (figures 4 and 5).

### 4.1. The initial viral load of the index case affects the rate at which the system converges to a steady state

To simulate disease spread, we introduced a multi-scale model describing transmission of a virus in a well-mixed, susceptible population. We described a within-host model with viral load dynamics dependent on the size of an initial viral load. Infected individuals had their transmission potential scaled by their viral load via a sigmoidal relationship. Additionally, the initial viral load of an infectee was scaled by the viral load of their infector at transmission, also via a sigmoid function. The influence of the infection dynamics of the index case(s) on outbreak development has been explored in similar simulation studies [1,28]. Using our model of disease transmission, we demonstrated *V*(0)_index_ affects the rate at which a population-level convergence process occurs, during which the distributions of initial viral loads in each subsequent generation converge to a steady state (figure 3).

The shape of the distribution of initial viral loads during the convergence of transmission dynamics reflects key aspects of the underlying model of viral transmission (figure 3). The emergence of the positively skewed distribution of initial viral loads in the infected population reflects the tendency for cases with higher initial viral loads to be more infectious, due to their increased peak viral loads. Once cases with high initial viral loads (i.e. *V*(0) > 400) appear during an outbreak, they will typically begin to dominate the infected population (see generations 2–5 in figure 3*a*). This process is reflected in the logistic shape of the convergence trajectories of the scenarios with low *V*(0)_index_ (see *V*(0)_index_ = 4.5, 14.2, 45.0 in figure 3*b*). In these scenarios, the distribution of initial viral loads in early generations are substantially different to the converged generation *g*_*c*_. The distribution of initial viral loads transitions to the converged distribution once cases with high initial viral loads appear and begin to dominate.

### 4.2. The initial viral load of the index case affects the accuracy of transmission parameter estimates

Having identified that *V*(0)_index_ could exert a substantial influence on the development of an outbreak, we then investigated its effect on estimation of transmission properties. We estimated the serial interval distribution and *R*_0_ across the first 50 days of outbreaks seeded with index cases with varying initial viral loads. We observed that when outbreaks were seeded with low initial viral load cases, the serial interval was typically overestimated and *R*_0_ was typically underestimated over the course of the early phase of the outbreak. This result has a clear correspondence with our analysis of the convergence of transmission dynamics for scenarios with lower *V*(0)_index_. Specifically, our finding that there would probably be an extended period where the infection dynamics of cases were not representative of later dynamics is clearly reflected in the tendency to overestimate the serial interval and underestimate *R*_0_ early in an outbreak.

Other analyses of outbreak data have found that if early transmission of a disease occurs in a subpopulation with a higher or lower intrinsic transmission rate, then the early estimates for the reproduction number will be biased [1]. In these circumstances, the subpopulation with a higher or lower intrinsic transmission rate is over-represented in the early observations of an outbreak; for example, an outbreak of influenza (which is typically more transmissible among children [33]) seeded in a school environment, where younger individuals make up a high proportion of the population. In this study, we demonstrated an alternative way that cases with a lower intrinsic transmission rate could emerge early in an outbreak, based solely on the mechanistic features of transmission in our multi-scale model and how such cases could then lead to misleading transmission property estimates. Specifically, as opposed to subpopulations defined on host characteristics such as age or gender, we modelled the initial viral load acting as a driver of transmission potential and correlating between generations. When outbreaks were seeded by cases with lower initial viral loads, we observed an early over-representation of cases with low initial viral loads which lead to lower reproduction number estimates, relative to those estimated after convergence.

In this work, we demonstrated the potential for reproduction number estimates to dynamically increase due to a transmission process which produces strong correlations in transmission dynamics between generations. We note that this particular result (a pronounced dynamic increase) depends on the choices we made in crafting our model of host-to-host transmission. For example, we chose to assume that higher viral loads produce a higher probability of transmission. While this is a biologically reasonable assumption [18], it ignores the possibility that behavioural responses could limit contact patterns due to the expression of severe symptoms associated with high viral loads (endogenous behaviour). An alternative model could suppose that low viral loads are in fact more likely to produce transmission, in which case the converged distribution of initial viral loads would be skewed to lower values. In such a case, the dynamic trend could be inverted, that is, if the index case has a viral load that is much higher than that of the average individual in the converged state, we would expect the later reproduction number estimates to decrease from their initial values. We suppose that the dynamics produced by correlations in viral load trajectories between generations will depend strongly on both the manifestation of disease, and the endogenous behavioural response of infected (and susceptible) individuals.

### 4.3. Misleading estimates could be operationally significant

We investigated the operational significance of misleading early estimates of the reproduction number. To do so, we analysed the relative bias (equation (2.11)) and confidence (equation (2.12)) associated with estimates of the reproduction number across the 50-day observation period (figures 4 and 5). We observed a central phase of the observation window (between days 30 and 40) where there was a potential for a misleading estimate to be produced with a substantial degree of confidence. In this case, underestimation of the reproduction number could have a serious impact on the perception of a risk in a community, and associated public health decisions. To highlight the potential for these estimates to be problematic, we assessed how the estimate could be used to forecast future outbreaks of the same pathogen in similar communities. Using a standard SIR compartmental model without vital dynamics, we adjusted the recovery rate and transmission rate parameters to compare how modelling would differ using the central *R*_0_ estimates at day 26 (*R*_0_ = 1.96), day 38 (*R*_0_ = 3.68) and day 50 (*R*_0_ = 4.02) of the representative outbreak sample shown in figure 4 (see electronic supplementary material, S11). Compared with the earlier day 26 estimate, using the day 38 and day 50 estimates resulted in an increase of approximately 155% and 176% in the peak number of infected individuals and a decrease of 58.3% and 62.5% in the time taken to reach the peak, respectively. The size and timing of the peak number of infected individuals are critical measures in planning public health responses and the difference shown here demonstrates the importance of the underestimation for policy decision makers.

Furthermore, media coverage of an emerging pathogen has a profound effect on the perception of a disease in a population, as has been witnessed in the current COVID-19 pandemic [34–36]. Misleading estimates, when reported to the public, have the potential to induce a sense of panic (overestimation) or complacency (underestimation) in public perception of risk due to a pathogen. Media coverage plays a role, alongside public policy formulation, in determining the public response to an outbreak which can have substantial effects on disease spread. The perception of a pathogen can also have serious effects on the economic output and mental well-being of a community [34].

### 4.4. Limitations and future work

Some key assumptions of the underlying outbreak model should be kept in mind when interpreting the results of our study. The determination of initial viral load in an infected individual and its resulting effect on disease progression are by no means established relationships in the study of viruses. Experimental data related to the initial viral load are difficult to obtain, mainly due to the difficulty of measuring early infection dynamics and the establishing dose in infected hosts. Similarly, the relationship between host viral load and infectiousness is not well characterized [18]. Most studies assume some positive relationship between the two quantities, typically with a linear, logarithmic or sigmoidal shape [18,22,23]. A possible avenue for future work would be to investigate the potential for misleading transmission property estimation to emerge from alternative representations of transmission.

Similarly, the assumption that case incidence is perfectly observed at symptom onset should be reflected upon when interpreting these results. The nature of the reproduction number estimation method means, if we have a consistent proportion of unobserved cases, we should still obtain accurate estimates. However, the likelihood of this proportion remaining steady across an outbreak is low [2]. Furthermore, the heterogeneous viral loads across the infected cases in our model may impact the likelihood of detecting some cases. Specifically, if severity of symptoms was positively related to the viral load of a case and the likelihood of observation was related to the presence of symptoms, it may mean lower viral load cases are less likely to be detected. Similarly, if test sensitivity was related to the viral load of a case, cases with lower viral loads may be poorly detected. An interesting extension of our model could be to see how relating likelihood of detection to viral load affects the quality of the estimates produced from the estimation procedures discussed. Additionally, the assumption that symptom onset occurs at peak viral load is not accurate for a variety of pathogens [37,38]. Altering our model to assume symptom onset occurs earlier at some fixed portion of the peak viral load is unlikely to affect the serial intervals observed in the converged outbreak setting (see electronic supplementary material, S16).

Another avenue for further work is to investigate how the outbreak dynamics explored in this analysis would play out in a later phase of pathogen emergence, where multiple outbreaks have been observed in different settings. One could explore how the composition of the initial conditions describing a set of simulated observed outbreaks affects the aggregate transmission property estimates. The challenge in this scenario is effectively incorporating data from different outbreaks together to produce one descriptive estimate. Hierarchical models have been used effectively to estimate disease parameters across different environments [39].

Finally, the heterogeneity in infectiousness between cases within our transmission model, due to varying viral loads, could lead to superspreading (i.e. a small proportion of cases in the infected population being substantially more infectious) [40]. Related work that considered similar transmission mechanisms to our study in the context of superspreading, suggests correlation of superspreaders (i.e. superspreaders being more likely to produce other superspreaders) may produce a delayed increase in outbreak growth [40]. While not defining particular hosts as superspreaders, we certainly observed in our study the delayed outbreak growth they suggest could occur due to this transmission mechanism. Further analysis of our model in the context of superspreading is another potential avenue for future work.

### 4.5. Conclusion

In summary, we developed a stochastic model of disease transmission, accounting for the transfer of virus from infected to susceptible hosts through the use of multi-scale modelling. We found the index case initial conditions were influential in the development of an outbreak. Finally, we demonstrated the potential for misleading estimates of the serial interval and *R*_0_ in the first 50 days of an outbreak. This investigation illustrates how multi-scale models—that explicitly capture the relationship between host-level viral dynamics and population-level transmission dynamics—can be used to evaluate widely used methods for estimating transmission properties.

## Data Availability

All analyses were performed in python3 & R. Data and code is available at https://doi.org/10.5281/zenodo.7735813. The data are provided in electronic supplementary material [41].
